# Identification of MEG8/miR‐378d/SOBP axis as a novel regulatory network and associated with immune infiltrates in ovarian carcinoma by integrated bioinformatics analysis

**DOI:** 10.1002/cam4.3854

**Published:** 2021-03-19

**Authors:** Jian Lei, Zhen‐Yu He, Jun Wang, Min Hu, Ping Zhou, Chen‐Lu Lian, Li Hua, San‐Gang Wu, Juan Zhou

**Affiliations:** ^1^ Department of Obstetrics and Gynecology the First Affiliated Hospital of Xiamen University Xiamen People’s Republic of China; ^2^ Department of Radiation Oncology Sun Yat‐sen University Cancer Center State Key Laboratory of Oncology in South China Collaborative Innovation Center of Cancer Medicine Guangzhou People’s Republic of China; ^3^ Department of Radiation Oncology the First Affiliated Hospital of Xiamen University Xiamen People’s Republic of China

**Keywords:** bioinformatics analysis, competing endogenous RNA, differently expressed genes, ovarian cancer, tumor‐infiltrating lymphocytes

## Abstract

**Background:**

To investigate the potential molecular mechanism of ovarian cancer (OC) evolution and immunological correlation using the integrated bioinformatics analysis.

**Methods:**

Data from the Gene Expression Omnibus was used to gain differentially expressed genes (DEGs). Gene Ontology and Kyoto Encyclopedia of Gene and Genome pathway analysis were completed by utilizing the Database for Annotation, Visualization, and Integrated Discovery. After multiple validations via The Cancer Genome Atlas (TCGA), Genotype‐Tissue Expression (GTEx) projects, the Human Protein Atlas, Kaplan–Meier (KM) plotter, and immune logical relationships of the key gene SOBP were evaluated based on Tumor Immune Estimation Resource, and Gene Set Enrichment Analysis (GSEA) software. Finally, the lncRNAs‐miRNAs‐mRNAs subnetwork was predicted by starBase, TargetScan, miRBD, and LncBase, individually. Correlation of expression and prognosis for mRNAs, miRNAs, and lncRNAs were confirmed by TCGA, Gene Expression Profiling Interactive Analysis 2 (GEPIA 2), starBase, and KM.

**Results:**

A total of 192 shared DEGs were discovered from the four data sets, including 125 upregulated and 67 downregulated genes. Functional enrichment analysis presented that they were mainly enriched in cartilage development, pathway in PI3 K‐Akt signaling pathway. Lower expression of SOBP was the independent prognostic factor for inferior prognosis in OC patients. The downregulation of SOBP enhanced the infiltration levels of B cells, CD8+ T cells, Macrophage, Neutrophil, and Dendritic cells. GSEA also disclosed low SOBP showed a significantly associated with the activation of various immune‐related pathways. Finally, we first reported that the MEG8/miR‐378d/SOBP axis was linked to the development and prognosis of OC through regulating the cytokines pathway.

**Conclusions:**

Our study establishes a novel MEG8/miR‐378d/SOBP axis in the development and prognosis of OC, and the triple subnetwork probably affects the progression of the OC by regulating the cytokines pathway.

## Graphical abstract

Our study establishes a novel MEG8/miR‐378d/SOBP axis in the development and prognosis of ovarian cancer, and the triple sub‐network probably affects the progression of ovarian tumor by regulating cytokines pathway.

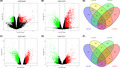

## 1 INTRODUCTION

Ovarian cancer (OC) is the eighth common cancer in females and the second cause of gynecologic tumor death. Globally, 295,414 (1.6% of all cancer cases) new OC patients and 184,799 (1.9% of all cancer cases) deaths occurred in 2018.^1^ Most OC patients were diagnosed with advanced‐stage tumor (stage III or IV), and surgery and platinum/taxane chemotherapy are still the main treatment for OC patients.^2^ However, over 75% of women with advanced‐stage OC will die from disease recurrence.^3^ Therefore, it is meaningful to investigate early screening biomarkers and potential therapeutic targets to improve outcomes of OC patients.

Tumor‐infiltrating lymphocytes (TILs) play a crucial role in the immune responses of human cancers.^4^ The certain antigens in the cell membrane divided TILs into several subsets, and each of the subsets has different functions in the development of malignant tumors. Several studies have reported that TILs improved prognosis in various types of malignant tumors, such as breast, lung, and colon cancer.^5^, ^6^, ^7^ In addition, several studies have also shown a distinct correlation among different TIL intensities regarding the survival outcomes.^8^, ^9^, ^10^ However, the clinical effect of immune cells in OC patients remains unclear. Therefore, the role of TILs in treatment decision making and prognostic assessment deserves further study.

The competing endogenous RNA (ceRNA) hypothesis was introduced by Salmena et al. in 2011, which described a brand‐new regulatory mechanism among ncRNAs and mRNAs.^11^ CeRNA hypothesis sustained that lncRNAs and mRNAs could cross talk by competitively binding to common miRNAs to achieve their biological functions.^12^ But the characters of the lncRNA‐miRNA‐mRNA network in OC were rarely explored. In addition, the potential mechanism of action and relationship with immunity in the ceRNA regulatory network is also worth exploring.

This study aimed to identify useful signature genes that were related to the development, metastasis, and prognostic values of OC, and investigated the underlying mechanism of hub genes through a comprehensive bioinformatics approach. First, we combined four gene expression sets (GSE36668, GSE12470, GSE14407, and GSE27651) from Gene Expression Omnibus (GEO) to obtain common differentially expressed genes (DEGs). Second, Gene Ontology (GO) and Kyoto Encyclopedia of Gene and Genome (KEGG) pathway analysis were utilized to search the function of the common DEGs in OC. Third, six hub genes (RIPK4, SCNN1A, SLC4A11, ELF3, CLDN4, and SOBP) were found after confirmation of the expression and survival of multiple databases. Especially, lower expression of the SOBP gene was independently associated with inferior survival outcomes in OC patients. In addition, high levels of immune infiltration and activation of immune pathways were associated with the downregulation of SOBP. Finally, we predicted the MEG8/miR‐378d/SOBP (lncRNA‐miRNA‐mRNA) axis using integrated bioinformatics analysis, which was associated with the development and prognosis of OC by regulating immune responses in the cytokines pathway.

## 2 METHODS AND MATERIALS

### 2.1 Differential expression analysis

We found four ovarian tumor data sets (GSE36668, GSE12470, GSE14407, and GSE27651) from the GEO database (http://www.ncbi.nlm.nih.gov/geo).^13^ The detailed data set information of the four OC data sets are shown in Table 1. The DEGs were obtained and visualized by conducting affy, limma, and ggplot2 packages in R software (version 3.5.3). |log2FC| >1.5 and adjusted *p*‐value <0.05 means statistically significant. VENNY (http://bioinfogp.cnb.csic.es/tools/venny/index.html) 2.1.0 was applied to get common DEGs and draw the Venn diagrams.

**TABLE 1:** Details for data sets from GEO

GEO	Platform	Sample	Normal	Tumor	Submission	Update	Author
GSE36668	GPL570	Tissue	4	4	21 Mar 2012	25 Mar 2019	Elgaaen BV
GSE12470	GPL887	Tissue	10	43	17 Aug 2008	06 Dec 2012	Yoshihara K
GSE14407	GPL570	Tissue	12	12	13 Jan 2009	25 Mar 2019	Bowen NJ
GSE27651	GPL570	Tissue	6	35	02 Mar 2011	25 Mar 2019	Wong K

Abbreviation: GEO, Gene Expression Omnibus.

### 2.2 Functional analysis of common DEGs

For a further understanding of the common DEGs, the Database for Annotation, Visualization, and Integrated Discovery (DAVID) was utilized to perform GO and KEGG enrichment analyses,^14^ and the results were visualized with ggplot2 and GO plot R packages. To identify the potential mechanism of SOBP on prognosis of OC, gene set enrichment analysis was conducted by Gene Set Enrichment Analysis (GSEA) software version 3.0.^15^ We divided 379 OC samples from The Cancer Genome Atlas (TCGA) into high SOBP group and low SOBP group by median value, other parameters criteria were set by default. Nominal *p* < 0.01, false discovery rate (FDR)<0.25, and |Normalized Enrichment Score (NES)| >1 were considered as statistically significant.

### 2.3 Gene expression profiling interactive analysis 2 (GEPIA2)

GEPIA2 (http://gepia2.cancer‐pku.cn) is an online tool for analyzing the transcriptional profiles of human cancers and normal tissues, by using the TCGA database and the Genotype‐Tissue Expression (GTEx) projects.^16^ We performed the analysis regarding the differential expression and prognostic values for common DEGs, and the relationship between MEG8 and SOBP was analyzed in the correlation analysis module. Genes with |log2FC| >1 and *p* < 0.05 were set as the cutoff criterion.

### 2.4 Kaplan–Meier (KM) plotter

The KM Plotter (http://kmplot.com/analysis/) was applied to verify the prognosis of the six key genes in OC.^17^ The KM Plotter could assess the influence of 54,675 genes on survival, including 6234 breast cancers, 3452 lung cancers, 2190 OC, and 1440 gastric cancers. The prognostic value of the mRNAs, miRNAs, and lncRNAs were showed with a hazard ratio (HR), 95% confidence interval (CI), and *p*‐value. The *p*‐value <0.05 was reflected statistically significant.

### 2.5 Immunohistochemistry

We used the Human Protein Atlas (https://www.proteinatlas.org/), which had mRNA and protein expression data on 44 different human normal tissues, as well as 17 common types of cancer.^18^ The antibody‐based protein data show the corresponding protein expression. The intensity of the staining was utilized to evaluate the six key genes in OC tissues at protein expression levels.

### 2.6 Oncomine

Oncomine (http://www.oncomine.org),^19^ an online database, was used to analyze the difference in mRNA expression of SOBP among normal tissues and cancer samples. The thresholds of *p*‐value and fold change (FC) were set as 0.001 and 1.5, respectively.

### 2.7 Tumor immune estimation resource (TIMER) analysis

To date, surgery and platinum/taxane chemotherapy are still the main treatment for OC patients.^2^ However, there is still no effective target index and drug application in the field of immunotherapy. Increasing evidence has indicated a close association between immune infiltration in cancer and clinical outcomes.^20^, ^21^ TIMER database (https://cistrome.shinyapps.io/timer/) was used to systematically analyze the tumor‐infiltrating immune cells in human cancers using more than 10,000 samples from the TCGA database.^22^ We explored the association between SOBP expression and the abundance of infiltrating immune cells. The marker genes used for the analysis were based on data from previous studies.^23^, ^24^ TIMER also output multivariate Cox analysis results, including hazard ratios and statistical significance. *p* < 0.05 was considered statistically significant.

### 2.8 Prediction and evaluation of upstream miRNAs and lncRNAs

Upstream miRNAs of SOBP were predicted by starBase, TargetScan, and miRBD, then, LncBase was applied to predict the upstream lncRNAs of miRNAs.^25^, ^26^, ^27^, ^28^ The common miRNAs were achieved by VENNY 2.1.0. To verify the relationship between miRNA and SOBP, we conducted correlation and survival analysis of common miRNAs in TCGA, starBase, and KM, individually. Similar methods were used to confirm the relationship between lncRNAs and hmiR‐378d. ImmLnc (http://bio‐bigdata.hrbmu.edu.cn), an online tool for identifying lncRNA regulators of immune‐related pathways, was using to explore the immunological relationship of MEG8. *p*‐value <0.05 was considered as statistically significant.

### 2.9 Statistical analyses

Ovarian normal and tumor tissue transcriptional profiles were obtained from GTEx and TCGA data sets across UCSC Xena Browser, including 88 ovarian tissues and 379 tumor samples. The Spearman correlation coefficient was utilized to calculate all expression correlations under SPSS 24.0 and pictured with the pheatmap package. The Student's *t*‐test was performed using GraphPad Prism 8.0. The related gene network was constructed in Cytoscape 3.6.0.^29^ *p*‐values <0.05 were considered to suggest statistical significance.

## 3 RESULTS

### 3.1 Identification of common DEGs

**FIGURE 1:**
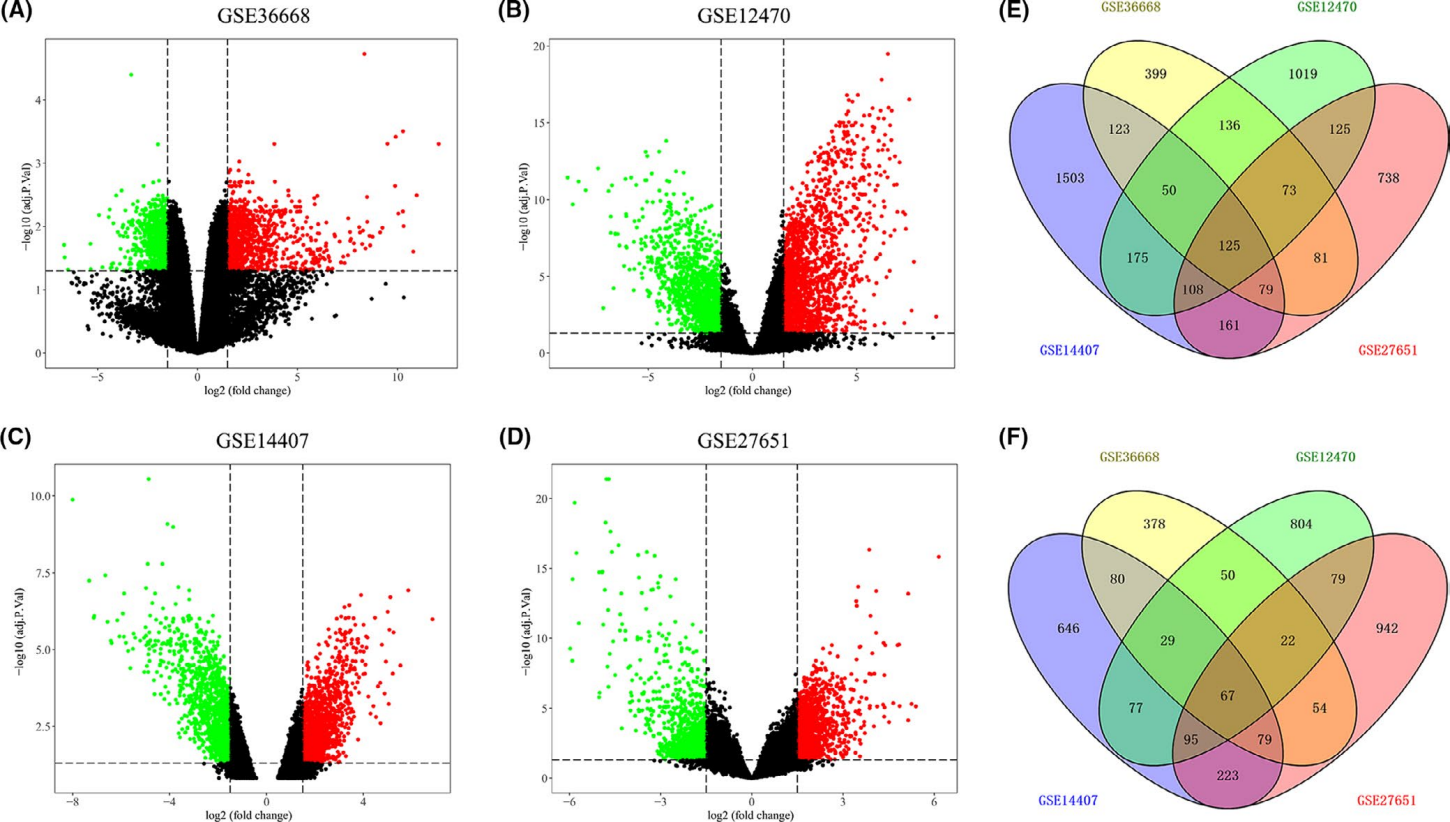
Differential expression genes in four data sets from GEO. (A–D) The volcano plots visualize the DEGs in GSE36668, GSE12470, GSE14407, and GSE27651, respectively. The red dots represent upregulated genes and green represent downregulated genes; (E) Venn diagram for the intersection of upregulated genes; (F) Venn diagram for the intersection of downregulated genes

After the analyses of GSE36668, GSE12470, GSE14407, and GSE27651 data sets, a total of 126 OC samples (94 tumor tissues and 32 normal tissues) were explored. There were 1825 DEGs screened from the GSE36668 data set, including 1066 upregulated and 759 downregulated genes; 3032 DEGs separated from the GSE12470 data set, including 1810 upregulated and 1222 downregulated genes; 3620 DEGs selected from the GSE14407 data set, including 2324 upregulated and 1296 downregulated genes. A total of 3051 DEGs were nominated from the GSE27651 data set, including 1490 upregulated and 1561 downregulated genes. The volcano plots of DEGs among each data set are shown in Figure 1A–D. By Venn diagram analysis, 192 common DEGs in the intersection of the four data sets were identified and selected for further analysis, containing 125 upregulation and 67 downregulation genes (Figure 1E and F).

### 3.2 Functional analysis of common DEGs

**FIGURE 2:**
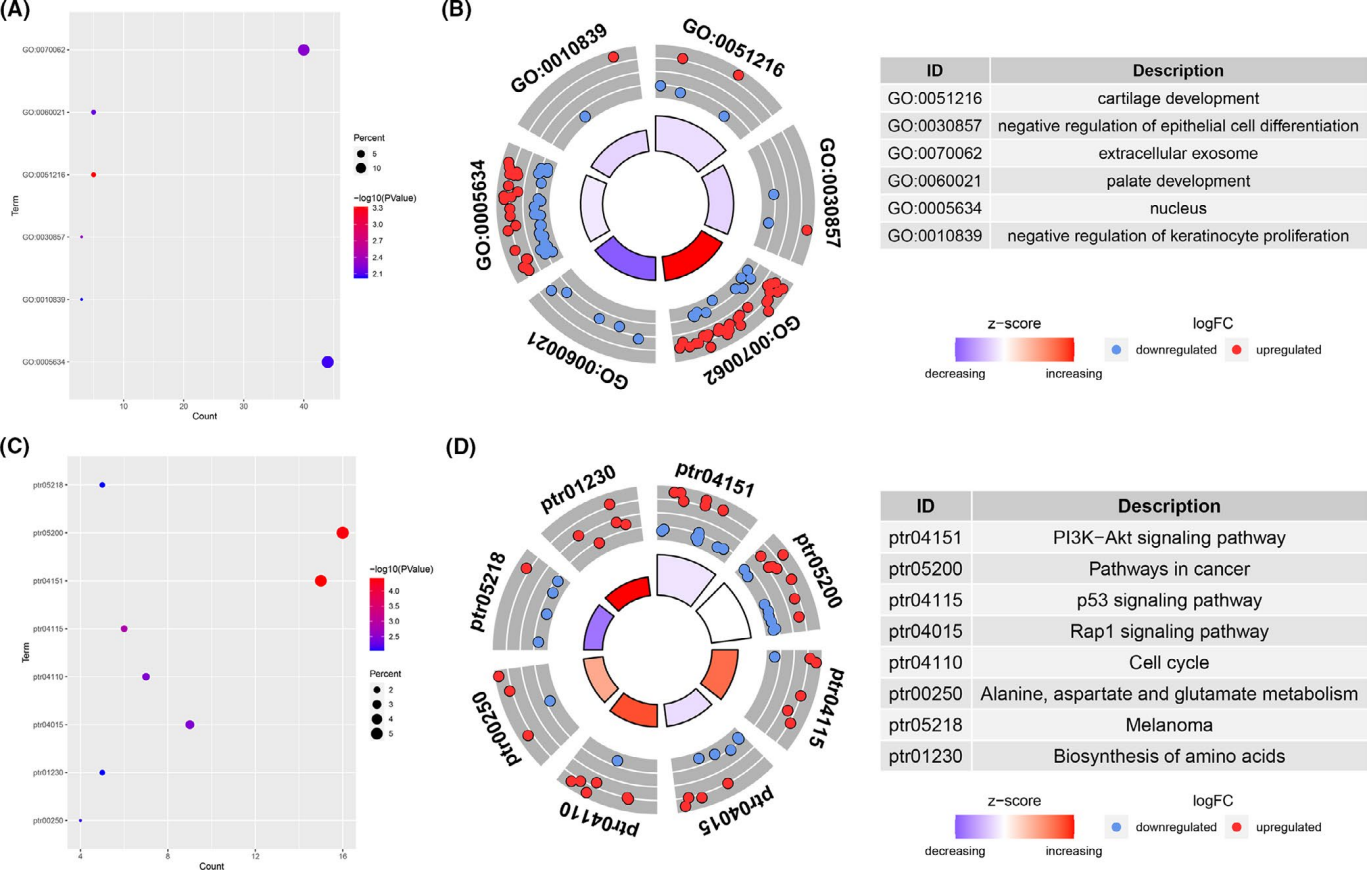
GO function and KEGG pathway analysis of 192 common DEGs. (A–B) GO analysis showed the function enrichment. (C–D) KEGG analysis showed the pathway enrichment

For a deeper understanding of these common DEGs, DAVID was applied to run GO and KEGG pathway enrichment analysis. GO analysis showed common DEGs were enriched in biological processes and cellular components (Figure 2A and B), including GO:0051216 (cartilage development), GO:0030857 (negative regulation of epithelial cell differentiation), GO:0060021 (palate development), GO:0010839 (negative regulation of keratinocyte proliferation), and GO:0070062 (extracellular exosome), GO:0005634 (nucleus). The pathways involved in common DEGs are shown in Figure 2C and D, including ptr04151 (PI3 K‐Akt signaling pathway), ptr05200 (Pathways in cancer), ptr04115 (p53 signaling pathway), ptr04015 (Rap1 signaling pathway), ptr04110(Cell cycle), ptr00250 (Alanine, aspartate and glutamate metabolism), ptr05218 (Melanoma), and ptr01230 (Biosynthesis of amino acids). The detailed statistics are presented in Table 2.

**TABLE 2:** GO function and KEGG pathway analysis of 192 common DEGs

Category	Term ID	Term description	Count	*p*‐value	FDR
Biological Progress	GO:0051216	cartilage development	5	4.78E−04	0.736
	GO:0030857	negative regulation of epithelial cell differentiation	3	4.53E−03	6.764
	GO:0060021	palate development	5	6.71E−03	9.876
	GO:0010839	negative regulation of keratinocyte proliferation	3	9.51E−03	13.721
Cellular Component	GO:0070062	extracellular exosome	40	5.45E−03	6.287
	GO:0005634	nucleus	44	8.55E−03	9.707
KEGG Pathway	ptr04151	PI3 K‐Akt signaling pathway	15	3.80E−05	0.046
	ptr05200	Pathways in cancer	16	4.40E−05	0.054
	ptr04115	p53 signaling pathway	6	1.43E−03	1.733
	ptr04015	Rap1 signaling pathway	9	3.58E−03	4.275
	ptr04110	Cell cycle	7	3.60E−03	4.290
	ptr00250	Alanine, aspartate and glutamate metabolism	4	7.29E−03	8.525
	ptr05218	Melanoma	5	9.17E−03	10.609
	ptr01230	Biosynthesis of amino acids	5	9.17E−03	10.609

### 3.3 Survival analysis

**FIGURE 3:**
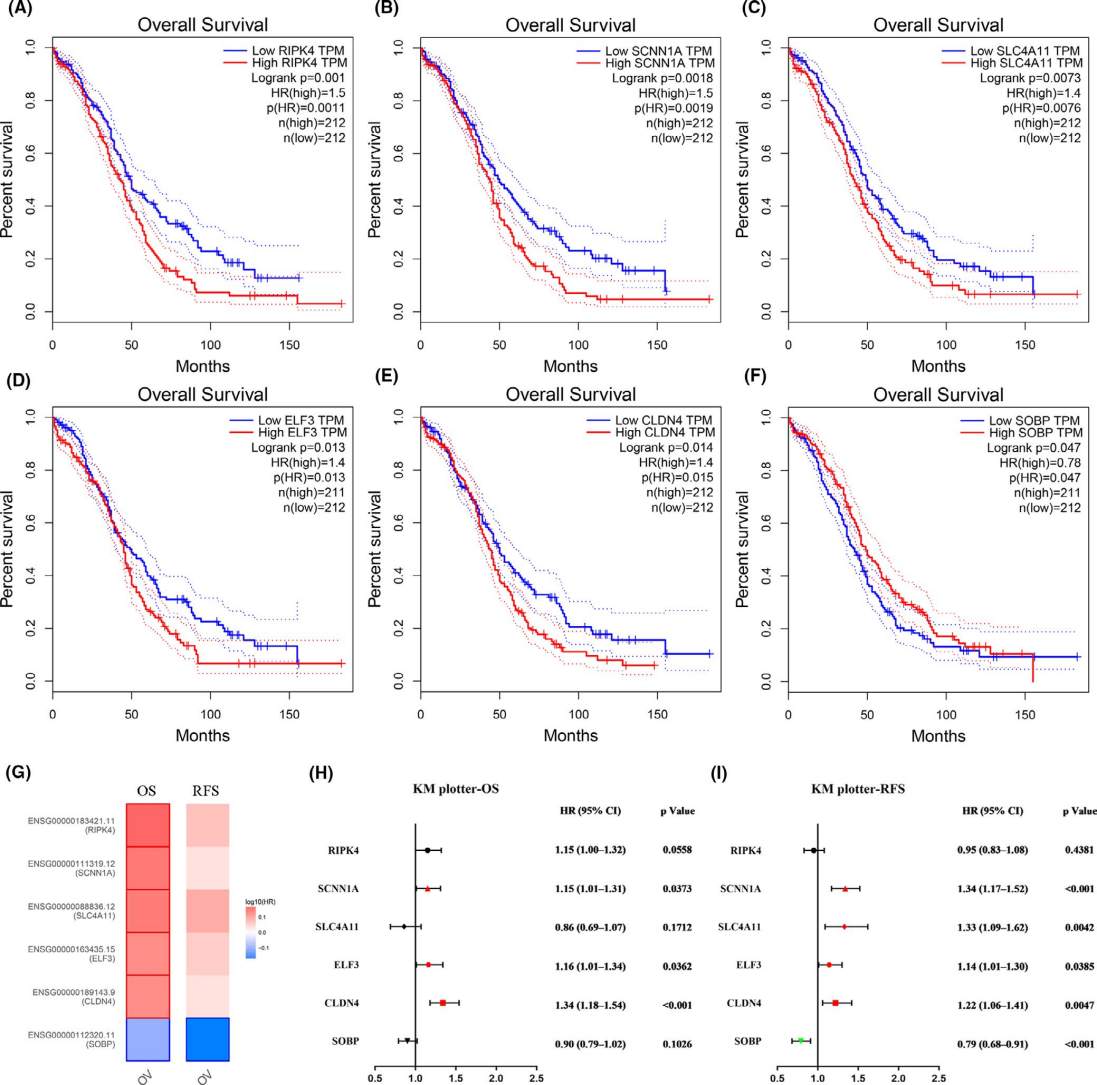
Prognostic values of the six hub genes (RIPK4, SCNN1A, SLC4A11, ELF3, CLDN4, and SOBP) in ovarian cancer. (A–F) Prognostic values of the six hub genes in overall survival (OS) from GEPIA2, separately; (G) a survival map of six genes for OS and relapse‐free survival (RFS) in ovarian cancer from GEPIA2; (H) prognostic values (OS) of six hub genes in from KM; (I) prognostic values (RFS) of six hub genes in from KM

To identify the key genes more accurately in OC, we explored the prognosis of the common DEGs by using GEPIA and KM, respectively. After the combined analyses of the expression and survival outcomes, we presented five upregulated key genes (RIPK4, SCNN1A, SLC4A11, ELF3, and CLDN4) were not only increased in OC samples, but also indicated poor overall survival (OS) in OC patients (Figure 3A–E and Figure 3G), and only one downregulated hub gene (SOBP) was associated with a lower OS (Figure 3F and G). As a validation, we performed survival analysis of the six candidate genes by using KM, only SOBP was associated with favorable relapse‐free survival (RFS) in both TCGA and KM data sets (Figure 3G–I). Moreover, SOBP was also an independent survival factor for OS, through a multivariable Cox proportional hazard model including designated clinical features and gene expression from TIMER (Table 3).

**TABLE 3:** Multivariate prognostic analysis of ovarian cancer patients using Cox proportional hazard model (TIMER)

Variables	HR	95%CI		*p*
		lower	upper	
Age	1.025	1.014	1.036	**<0.001**
Race (Black)	1.153	0.444	2.993	0.770
Race (White)	0.842	0.369	1.918	0.682
Purity	0.185	0.054	0.636	**0.007**
B_cell	1.670	0.002	1213.754	0.879
CD8_Tcell	0.330	0.007	14.723	0.567
CD4_Tcell	<0.001	<0.001	0.017	**0.002**
Dendritic	0.386	0.008	19.576	0.635
SOBP	0.597	0.417	0.856	**0.005**

Bold values indicate *p* < 0.01.

### 3.4 Expression validation of candidate genes

**FIGURE 4:**
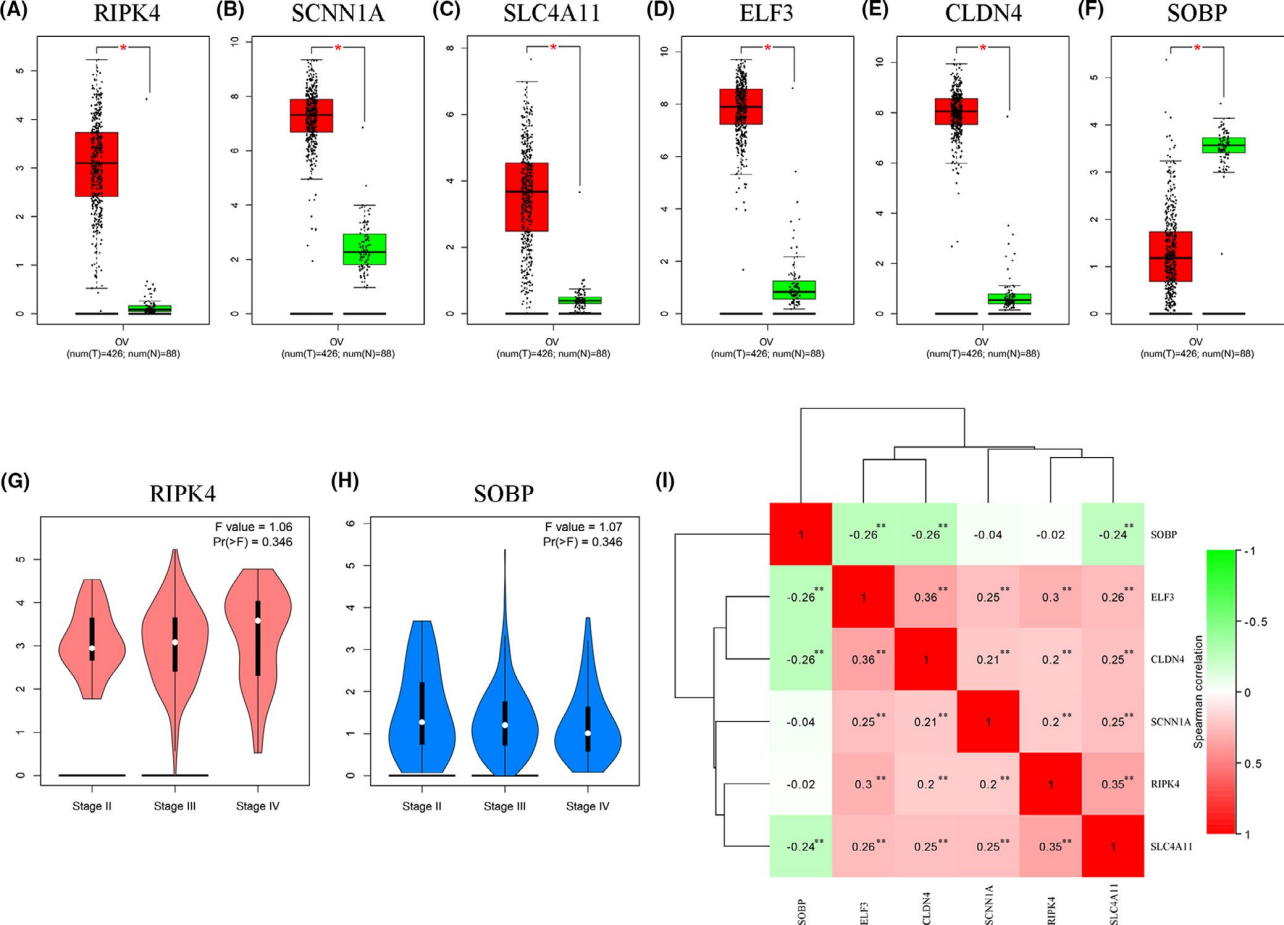
Validation of the mRNA expression of six hub genes from TCGA and GTEx ovarian cancer samples. (A–F) The mRNA expression of RIPK4, SCNN1A, SLC4A11, ELF3, CLDN4, and SOBP in ovarian normal tissues and cancer tissues; Red: tumor; Green: normal; (G) the relationship between RIPK4 expression and tumor stage; (H) the relationship between SOBP expression and tumor stage. (I) Correction between six hub genes in ovarian cancer (TCGA)

**FIGURE 5:**
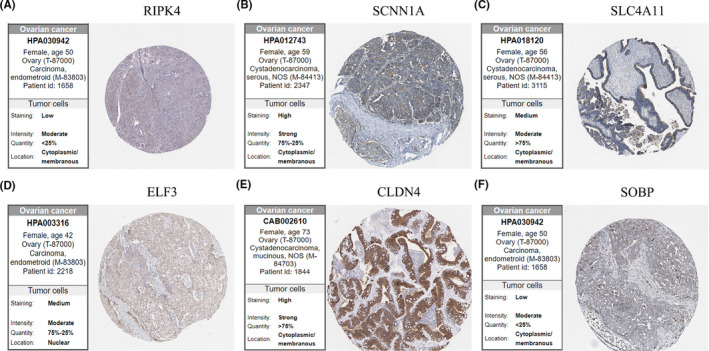
Representative immunohistochemistry images of six hub genes protein expression in ovarian cancer. (A) RIPK4; (B) SCNN1A; (C) SLC4A11; (D) ELF3; (E) CLDN4; (F) SOBP

Subsequently, TCGA OC samples and GTEx normal tissues were used to approve mRNA expression of six key genes. As shown in Figure 4, all the results of six genes (RIPK4, SCNN1A, SLC4A11, ELF3, CLDN4, and SOBP) were in accordance with previous data. Meanwhile, RIPK4 and SOBP mRNA expression was associated with the tumor pathological stage, which was consistent with their survival outcomes. The upregulated five genes showed a positive correlation with each other, while the expression of SOBP was negatively correlated with ELF3, CLDN4, and SLC4A11, significantly (Figure 4I). Representative immunohistochemistry images of six gene protein expressions in OC tissues are shown in Figure 5.

### 3.5 Correlation between SOBP expression and infiltration level of immune cells

**FIGURE 6:**
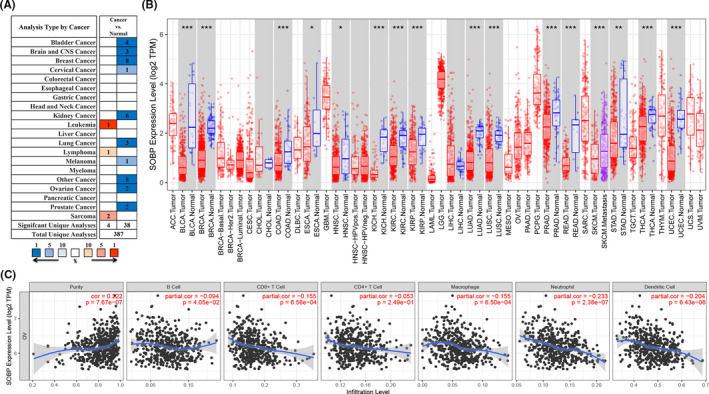
The mRNA of SOBP in human cancer and related infiltration levels of immune cells in ovarian cancer. (A) SOBP expression in human tumors compared with normal tissues (Oncomine); (B) the SOBP expression in human cancers from TCGA samples (TIMER); *p‐value<0.05, **p<0.01, ***p<0.001; (C) correlation analysis of the SOBP expression and infiltration levels of immune cells in ovarian cancer (TIMER)

Our findings showed that SOBP was a hub gene in OC in combination with the results of mRNA, protein expression levels, and prognostic assessment. Furthermore, we analyzed the relationship between SOBP expression and immune cell infiltration via the TIMER database. We performed a pan‐cancerous analysis of mRNA expression by using Oncomine and TIMER databases, the results showed SOBP was a lower expression in various human cancers, including OC (Figure 6A and B). We then noticed that low SOBP expression correlated with elevated infiltration of generally immune cell types in OC and showed a significant correlation with tumor purity. Specifically, the lower expression level of SOBP was correlated with the high infiltration of CD8+ T cells (*r* = −0.155, *p* = 6.56e‐04), Macrophages (*r* = −0.155, *p* = 6.50e‐04), Neutrophils (*r* = −0.233, *p* = 2.38e‐07), and Dendritic cells (*r* = −0.204, *p* = 6.43e‐06) in OC tissues (Figure 6C).

Next, we investigated the correlation among the transcription of SOBP and the rank of tumor‐infiltrating immune cells in more detail, based on the immune marker genes expression in OC tissues from the TIMER databases. The results showed that the mRNA of SOBP was inversely related to the expression of marker genes of designated immune cells, including CD8+ T cell, T cell (general), Monocyte, TAM (tumor‐associated macrophage), M1 Macrophage, M2 Macrophage, Neutrophils, Natural killer cells, Dendritic cells, Th 1 (T helper cell 1), Treg (regulatory T cell), and T cell exhaustion (Table 4).

**TABLE 4:** Correlation analysis between SOBP and marker genes of immune cells (TIMER)

Description	Gene markers	None		Purity	
		R	*p*	R	*p*
CD8+ T cell	CD8A	−0.147	*	−0.159	*
	CD8B	0.011	0.842	0.016	0.807
T cell (general)	CD3D	−0.257	***	−0.311	***
	CD3E	−0.202	***	−0.260	***
	CD2	−0.217	***	−0.264	***
B cell	CD19	0.048	0.407	0.093	0.145
	CD79A	−0.067	0.242	−0.046	0.473
Monocyte	CD86	−0.192	***	−0.271	***
	CD115(CSF1R)	−0.084	0.146	−0.179	**
TAM	CCL2	−0.178	**	−0.209	***
	CD68	−0.137	*	−0.212	***
	IL10	−0.032	0.574	−0.022	0.731
M1 Macrophage	INOS(NOS2)	0.199	***	0.203	**
	IRF5	−0.021	0.715	−0.061	0.336
M2 Macrophage	COX2 (PTGS2)	0.149	**	0.135	*
	CD163	−0.076	0.188	−0.132	*
	VSIG4	−0.135	*	−0.204	**
	MS4A4A	−0.092	0.109	−0.159	*
Neutrophils	CD66b(CEACAM8)	0.163	**	0.131	*
	CD11b(ITGAM)	−0.092	0.109	−0.171	**
	CCR7	−0.033	0.565	−0.073	0.250
Natural killer cell	KIR2DL1	−0.066	0.255	−0.040	0.531
	KIR2DL3	−0.228	***	−0.259	***
	KIR2DL4	−0.259	***	−0.285	***
	KIR3DL1	−0.130	*	−0.147	*
	KIR3DL2	−0.019	0.738	−0.037	0.563
	KIR3DL3	−0.149	**	−0.150	*
	KIR2DS4	−0.134	*	−0.161	*
Dendritic cell	HLA‐DPB1	−0.228	***	−0.259	***
	HLA‐DQB1	−0.195	***	−0.185	**
	HLA‐DRA	−0.251	***	−0.249	***
	HLA‐DPA1	−0.237	***	−0.249	***
	BDCA−1(CD1C)	−0.060	0.297	−0.120	0.059
	BDCA−4(NRP1)	0.136	*	0.087	0.169
	CD11c(ITGAX)	−0.081	0.157	−0.150	*
Th1	T‐bet(TBX21)	−0.177	**	−0.256	***
	STAT4	−0.023	0.693	−0.042	0.513
	STAT1	0.014	0.803	0.060	0.346
	IFN‐y(IFNG)	−0.173	**	−0.160	*
	TNF‐a(TNF)	−0.121	*	−0.094	0.140
Th2	GATA3	−0.002	0.968	0.002	0.974
	STAT6	0.089	0.121	0.068	0.284
	STAT5A	−0.048	0.408	−0.121	0.057
	IL13	−0.072	0.210	−0.051	0.427
Tfh	BCL6	0.063	0.277	−0.007	0.908
	IL21	−0.054	0.345	−0.056	0.383
Th17	STAT3	0.063	0.273	0.021	0.746
	IL17A	−0.071	0.216	−0.095	0.137
Treg	FOXP3	−0.107	0.063	−0.117	0.066
	CCR8	−0.165	**	−0.156	*
	STAT5B	0.326	***	0.263	***
	TGFβ(TGFB1)	0.061	0.289	−0.006	0.920
T cell exhaustion	PD−1(PDCD1)	−0.096	0.095	−0.115	0.069
	CTLA4	−0.165	**	−0.170	**
	LAG3	−0.148	**	−0.161	*
	TIM−3(HAVCR2)	−0.203	***	−0.292	***
	GZMB	−0.280	***	−0.291	***

TAM, tumor‐associated macrophage; R, Spearman rank correlation coefficient; None, correlation without adjustment. Purity, correlation adjusted by purity.

* *p* < 0.05.

** *p* < 0.01.

*** *p* < 0.001.

### 3.6 Gene set enrichment analysis

**FIGURE 7:**
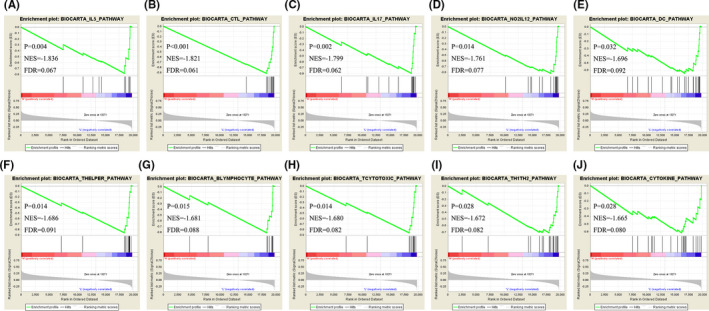
Gene set enrichment analysis of SOBP in the TCGA ovarian cancer samples

Further investigation of the biological signal pathways of SOBP in OC was performed in a single gene GSEA. The results showed various immune‐related pathways were upregulated in low SOBP expression group, including IL 5 signaling pathway (*p* = 0.004, NES = −1.836, FDR = 0.067), CTL (Cytotoxic T lymphocytes)‐mediated immune response against target cells (*p* < 0.001, NES = −1.821, FDR=0.061), IL 17 signaling pathway (*p* = 0.002, NES = −1.799, FDR = 0.062), NO2‐dependent IL 12 Pathway in NK cells (*p* = 0.014, NES = −1.761, FDR = 0.077), dendritic cells in regulating TH1 and TH2 development (*p* = 0.032, NES = −1.696, FDR = 0.092), T helper cell surface molecules (*p* = 0.014, NES = −1.686, FDR = 0.091), B lymphocyte cell surface molecules (*p* = 0.015, NES = −1.681, FDR = 0.088), T cytotoxic cell surface molecules (*p* = 0.014, NES = −1.680, FDR=0.082), Th1/Th2 differentiation (*p* = 0.028, NES = −1.672, FDR = 0.082), and cytokine network (*p* = 0.028, NES = −1.665, FDR = 0.080) (Figure 7A–J).

### 3.7 Construction of MEG8/miR‐378d/SOBP triple subnetwork

**FIGURE 8:**
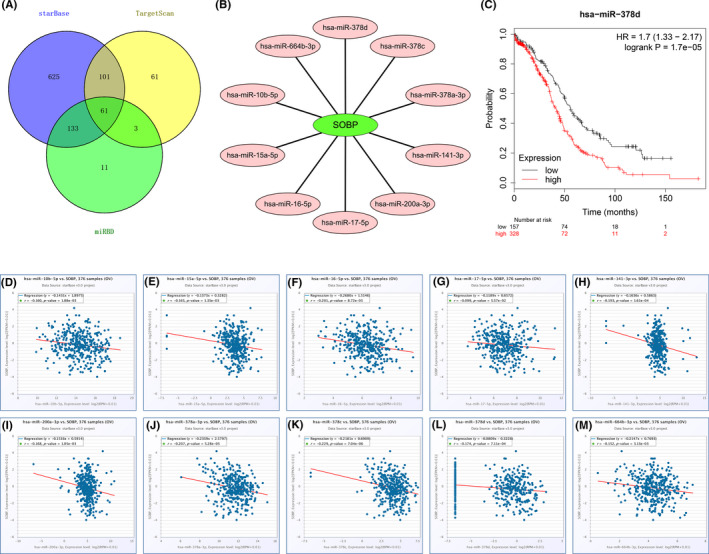
Identification of upstream potential miRNAs of SOBP in ovarian cancer. (A) The potential miRNAs of SOBP predicted by starBase, TargetScan, and miRBD; (B) the miRNAs‐SOBP network constructed by Cytoscape. (C) The prognosis (OS) of hsa‐miR‐378d from KM. (D–M) The expression correlation of key miRNAs and SOBP in OC

**FIGURE 9:**
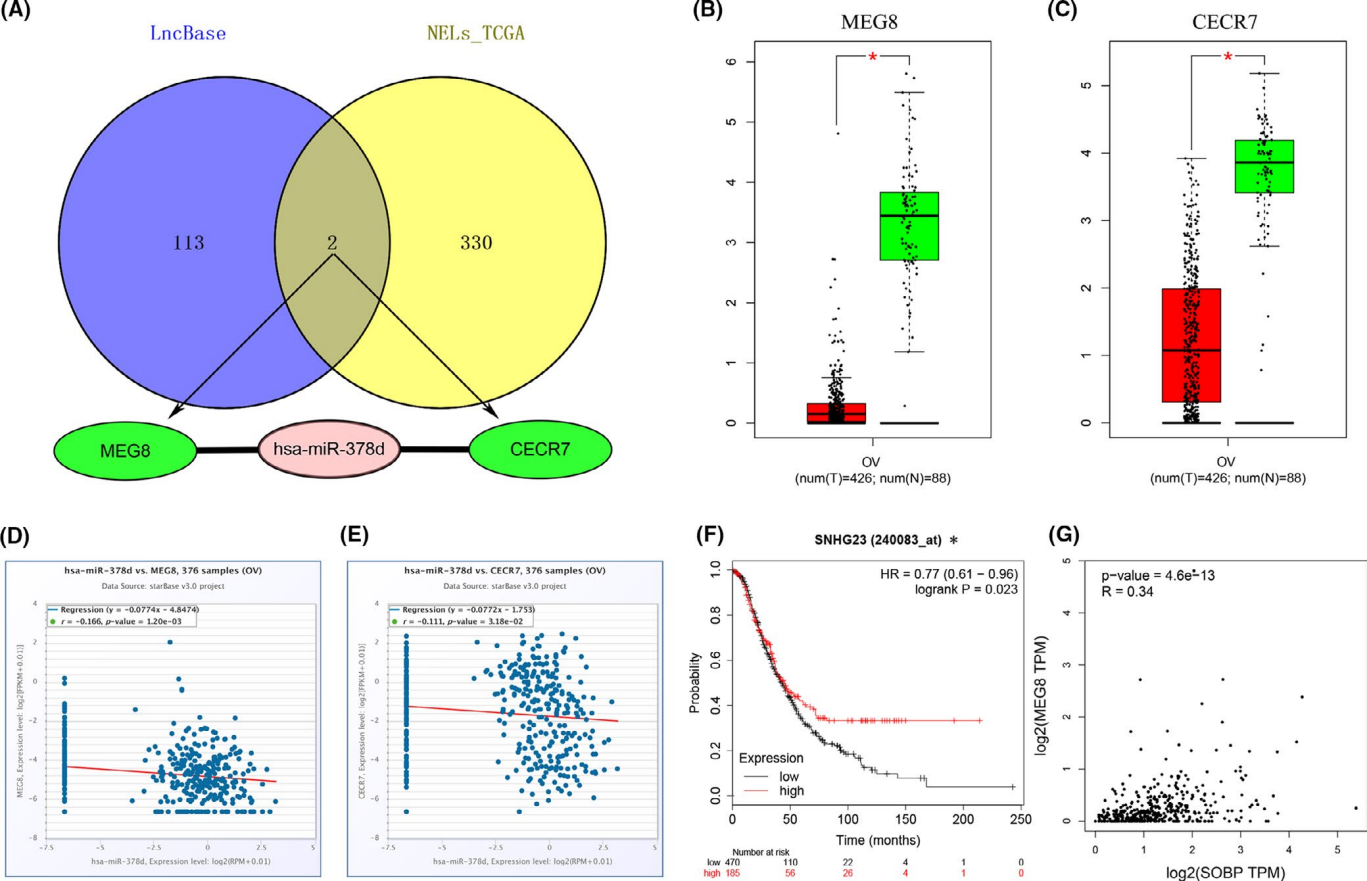
Screening the significant lncRNAs of hsa‐miR‐378d in ovarian tumor. (A) Venn diagram for the intersection of underlying lncRNAs from LncBase and negatively expressed lncRNAs (NELs) from TCGA; (B) the expression levels of lncRNA‐MEG8 in ovarian normal and cancer tissues (GEPIA2); (C) the expression levels of lncRNA‐CECR7 in ovarian normal and cancer samples (GEPIA2); (D) the expression correlation of lncRNA‐MEG8 and hsa‐miR‐378d in OC; (E) the expression correlation of key lncRNA‐ CECR7 and hsa‐miR‐378d in OC; (F) the prognostic value (OS) of lncRNA‐MEG8 from KM, *lncRNA‐MEG8 is also known as SNHG23; (G) the expression correlation of lncRNA‐MEG8 and SOBP in OC

By a series of upstream prediction and correlation evaluations in expression, a brand‐new lncRNA‐miRNA‐mRNA triple regulatory axis in OC was built. First, we predicted upstream miRNAs of SOBP in starBase, TargetScan, and miRBD separately and got 61 common miRNAs (Figure 8A). After approval of expression and survival analysis, 10 key miRNAs (hsa‐miR‐10b‐5p, hsa‐miR‐15a‐5p, hsa‐miR‐16‐5p, hsa‐miR‐17‐5p, hsa‐miR‐200a‐3p, hsa‐miR‐141‐3p, hsa‐miR‐378a‐3p, hsa‐miR‐378c, hsa‐miR‐378d, and hsa‐miR‐664b‐3p) were negatively correlated with the expression of SOBP in TCGA samples (Figure 8B, 8D–M), and only miR‐378d indicated poor prognosis (Figure 8C). Subsequently, we found the 115 upstream lncRNAs of miR‐378d from the LncBase database, and draw the Venn diagram with 332 Negative Expressed LncRNAs (NELs) for miR‐378d from TCGA analysis, only two lncRNAs (MEG8 and CECR7) showed predictability and negative correlation (Figure 9A). Equally, we verified the expression levels and prognostic values of two key lncRNAs. The results showed both of them were a lower expression in OC and negatively correlated with miR‐378d from GEPIA2 and starBase database, only the expression of lncRNA‐MEG8 was related to better OS in KM and higher SOBP in TCGA (Figure 9B–G). Altogether, the MEG8/miR‐378d/SOBP competing endogenous RNA (ceRNA) axis could be the potential mechanism in affecting tumor progression and prognosis of OC.

Finally, several immune pathways have been found to be associated with lncRNA‐MEG8 by using ImmLnc, including antigen processing and presentation, cytokine receptors, TGFb family member, cytokines, and natural killer cell cytotoxicity (Table 5). In particular, the pathway of cytokines also showed significant correlations with SOBP in the previous GESA report. Therefore, the cytokines pathway must be the most probable immune response mechanism of the MEG8/miR‐378d/SOBP axis in OC.

**TABLE 5:** Identification of the immune‐related pathways of lncRNA‐MEG8 in ovarian cancer

Category	Description	P Adjust	Marker Gene Number
Immune Pathway	Antigen Processing and Presentation	0.026	47
	Cytokine Receptors	0.026	82
	TGFb Family Member	0.026	15
	Cytokines	0.028	72
	Natural Killer Cell Cytotoxicity	0.028	40

## 4 DISCUSSION

Despite continued advances in treatment for OC, only 19% of patients were diagnosed at an early stage due to the lack of specific clinical symptoms and rapid cancer progression.^30^ Approximately 20–30% of patients would progress or developed disease recurrence in 6 months after completing chemotherapy and had a median OS of only 12–18 months.^31^ Therefore, investigating the effective biomarkers and identifying their effects in the diagnosis, development, and treatment of OC is a matter of urgency. In this study, we integrated the four expression data sets (GSE36668, GSE12470, GSE14407, and GSE27651) and obtained 192 common DEGs. GO analysis showed that common DEGs were enriched in cartilage development and negative regulation of epithelial cell differentiation, palate development, negative regulation of keratinocyte proliferation, extracellular exosome, and nucleus. KEGG pathways showed that the pathways regarding the abovementioned DEGs were significantly associated with the biological behavior of OC, including PI3 K‐Akt signaling pathway, Pathways in cancer, p53 signaling pathway, Rap1 signaling pathway, Cell cycle, Alanine, aspartate and glutamate metabolism, Melanoma, and Biosynthesis of amino acids.

After systematic expression and survival analysis, six genes (RIPK4, SCNN1A, SLC4A11, ELF3, CLDN4, and SOBP) were recognized as key genes that may be associated with the development of OC. Expression levels of RIPK4, SCNN1A, SLC4A11, ELF3, and CLDN4 were amplified and their upregulation was linked to poor prognosis of patients with OC. The five genes have been described to act as oncogenes in various human tumors and link to tumor progression. Moreover, several studies have also confirmed that the five genes may provide capable biomarkers for cancer. For example, RIPK4 expression is upregulated in nasopharyngeal, pancreatic, bladder urothelial, and cervical squamous cell carcinoma, and is associated with a poor outcome.^32^, ^33^, ^34^, ^35^ Increased SCNN1A contributed to unfavorite prognosis in pulmonary adenocarcinoma and OC.^36^, ^37^ Qin et al. also showed a high expression of SLC4A11 was an independent prognostic factor for poor OS in grade 3 or 4 serous OC through bioinformatics analysis.^38^ ELF3 overexpression is significantly linked to poor outcomes in hepatocellular, colorectal cancer, and lung adenocarcinoma patients, and that ELF3 enhance cell growth, migration in these cancers.^39^, ^40^, ^41^, ^42^ Based on the previous studies, CLDN4 showed high expression in many epithelial malignant tumors, such as ovarian and pancreatic cancer.^43^, ^44^, ^45^, ^46^ All these studies including ours showed that RIPK4, SCNN1A, SLC4A11, ELF3, and CLDN4 genes may be the five key oncogenes in the progression of OC. Regarding SOBP (sine oculis‐binding protein) gene (also named JXC1), it has rarely been reported in human diseases and has never been studied in tumors. Chen's results showed SOBP is crucial for cochlear growth, cell fate, and patterning of the organ of Corti.^47^ Birk et al. indicated that SOBP is altered in intellectual disability and is overexpression in the brain limbic system.^48^ In this study, we creatively explored the role of SOBP in OC patients. The results confirmed that SOBP was a low expression in mRNA and protein levels in OC tissues, and the downregulated of SOBP was related to poor OS and RFS in OC patients. Hence, SOBP could be considered a prognostic marker of OC, and potential mechanisms continue to be revealed.

Recently, the microenvironment including several kinds of cell bunches (such as tumor cells, immune cells, and fibroblasts) in the tumor has been a hot topic. According to the mRNA expression level of SOBP, we obtained nine types of tumor‐infiltrating lymphocytes in OC tissues, containing B cells, CD8+ T cells, Th 1, Treg, T cell exhaustion, macrophages (TAM, M1, and M2), neutrophils, dendritic, and natural killer cells from TIMER. The infiltration of these TILs was discovered to be inversely correlated with SOBP. Meanwhile, we identified 10 key upregulated pathways about the immune response in the low SOBP group, such as IL 5 signaling pathway, CTL (Cytotoxic T lymphocytes)‐mediated immune response against target cells, IL 17 signaling pathway, NO2‐dependent IL 12 Pathway in NK cells, Dendritic cells in regulating TH1 and TH2 development, T helper cell surface molecules, B lymphocyte cell surface molecules, T cytotoxic cell surface molecules, Th1/Th2 differentiation, and Cytokine network. These findings suggested that SOBP expression was strictly related to the level of TILs in OC tissues, and further analyses will focus on detailed mechanisms of SOBP modifying these infiltrating immune cells in OC. The intersection between SOBP expression and immune functions in OC implies a potential target of SOBP for the next treatment of OC patients. Meanwhile, the roles of lncRNAs have drawn more and more attention.^49^ Several studies indicated that lncRNAs may act as competitive endogenous RNA (ceRNAs) in a new triple regulatory network by competitively binding their shared miRNAs.^50^, ^51^, ^52^, ^53^ However, neither lncRNAs nor miRNAs have been described to engage in the direct modulation of SOBP in OC. In this work, we noticed the existence of various lncRNAs and miRNAs which may lead to aberrant expression of SOBP. Furthermore, by correlation of expression and prognosis, we assumed a regulatory axis of lncRNA‐miRNA‐mRNA must affect the development and progression of OC.

In our study, three databases (starBase, TargetScan, and miRBD) were used to predict miR‐378d as the upstream miRNA of SOBP simultaneously. The negative correlation occurred between the expression of miR‐378d and SOBP, and the high miR‐378d was associated with poor prognostic effect in OC. Similarly, lncRNA‐MEG8 could be regarded as the upstream of miR‐378d with a negative correlation in expression, and the lower MEG8 was bound up with an unfavorable prognosis in OC. Even more, the same related immune pathway, the cytokines pathway, was found among MEG8 and SOBP. In previous studies, miR‐378d was upregulated in colorectal cancer and might be a potential biomarker in diagnosis.^54^, ^55^ MEG8 suppresses activation of the epithelial–mesenchymal transition of hepatocytes by the Notch pathway.^56^ Therefore, under a comprehensive analysis and previous reports, we establish the MEG8/miR‐378d/SOBP axis and speculate that it may act a crucial role in the tumor progression and immune function of OC by regulating the cytokines pathway.

In general, we studied the differential expression of mRNA and protein levels in normal ovarian and tumor tissue samples. In addition, the key gene SOBP was creatively screened out for verification by combining the results of multiple databases. In addition, we reported the novel MEG8/miR‐378d/SOBP ceRNA subnetwork first, and speculate that it may act a crucial part in the tumor progression and immune function of OC by regulating the cytokines pathway. Thus, SOBP could offer great clinical value in the diagnosis, prognosis evaluation, and immunotherapy of OC patients.

There were several limitations in this study. First, we explored the expression of SOBP in OC using multiple public databases, and we did not prove these findings in our clinical samples. Second, further studies should be conducted to support the consistency with protein level and causality relation between different variables using western blot. Third, because this was a retrospective study, the level of evidence is imperfect. Although we creatively launched a novel lncRNA‐miRNA‐mRNA subnetwork in OC and predicted potential immune‐related pathways implicated in its function, more studies are warranted to elucidate the specific roles of MEG8/miR‐378d/SOBP and underlying mechanisms that affect tumor development and progression.

## 5 CONCLUSIONS

In summary, we detected six DEGs (RIPK4, SCNN1A, SLC4A11, ELF3, CLDN4, and SOBP) which can serve as tumor markers for the diagnosis and prognosis of OC. In particular, the gene SOBP was significantly low expressed in OC and negatively correlated with TILs. Moreover, a novel MEG8/miR‐378d/SOBP axis probably affects the progression of OC by regulating the cytokines pathway and may be utilized as a potential target of immunotherapy in the future. Even so, further biological function validation is required to reinforce this conclusion.

## CONSENT FOR PUBLICATION

Not applicable

## CONFLICT OF INTERESTS

The authors have no conflicts of interest to disclose.

## ETHICS APPROVAL

We analyzed the de‐identified information for patients contained in Gene Expression Omnibus and The Cancer Genome Atlas database; therefore, the present study was exempted from approval by the Institutional Review Board.

## Data Availability

The data that support the findings of this study are openly available in Gene Expression Omnibus (http://www.ncbi.nlm.nih.gov/geo) and University of California Santa Cruz Xena browser (UCSC Xena: http://xena.ucsc.edu/).
